# Supplementary material for: Characterizing and Removing Artifacts Using Dual-Layer EEG during Table Tennis
Source: Sensors (Basel). 2022 Aug 5;22(15):5867. doi: 10.3390/s22155867 (PMC9371038; doi:10.3390/s22155867)
Supplement: Supplementary file 1 [file sensors-22-05867-s001.zip › SupplementaryFigure_S1.pdf]

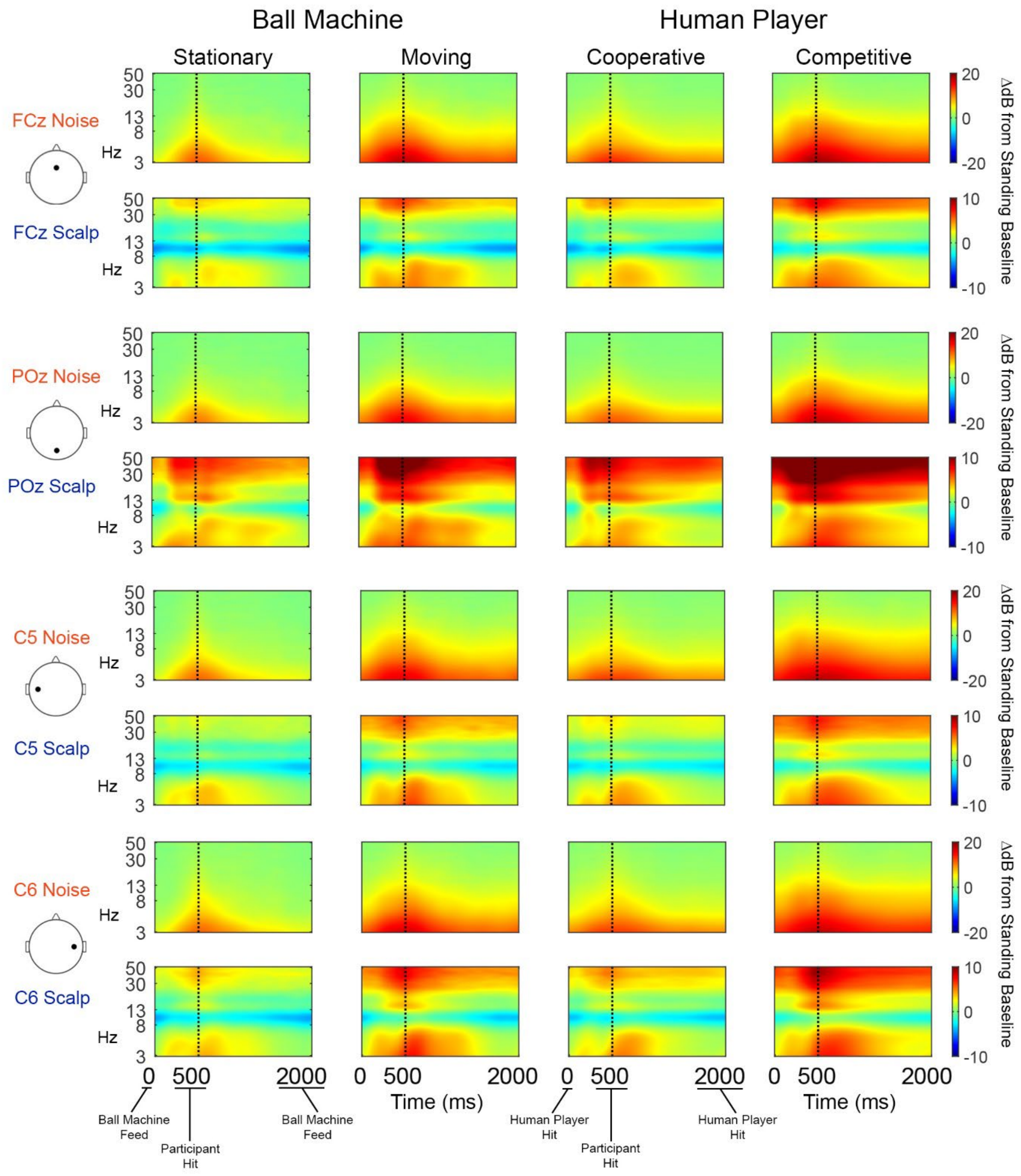

SUPPLEMENTARY FIGURE S1. **Unmasked group average event-related spectral perturbation (time-frequency) plots for the electrode pairs (n=20). Noise electrodes are in rows 1, 3, 5, and 7. Scalp-matched electrodes are shown in rows 2, 4, 6, and 8. Each hitting condition is shown in a different column. Increases in spectral power relative to standing baseline are in red and decreases in power relative to standing baseline are in blue. Vertical dashed lines indicate events in a single "swing cycle". We defined a "swing cycle" as 3 events: 1) when the ball was presented to the participant as a ball machine feed or human plyer hit, 2) when the participant hit the ball, and 3) when the ball was presented to the participant for the next hit.**
