# Supplementary material for: Characterizing and Removing Artifacts Using Dual-Layer EEG during Table Tennis
Source: Sensors (Basel). 2022 Aug 5;22(15):5867. doi: 10.3390/s22155867 (PMC9371038; doi:10.3390/s22155867)
Supplement: Supplementary file 1 [file sensors-22-05867-s001.zip › SupplementaryFigure_S3.pdf]

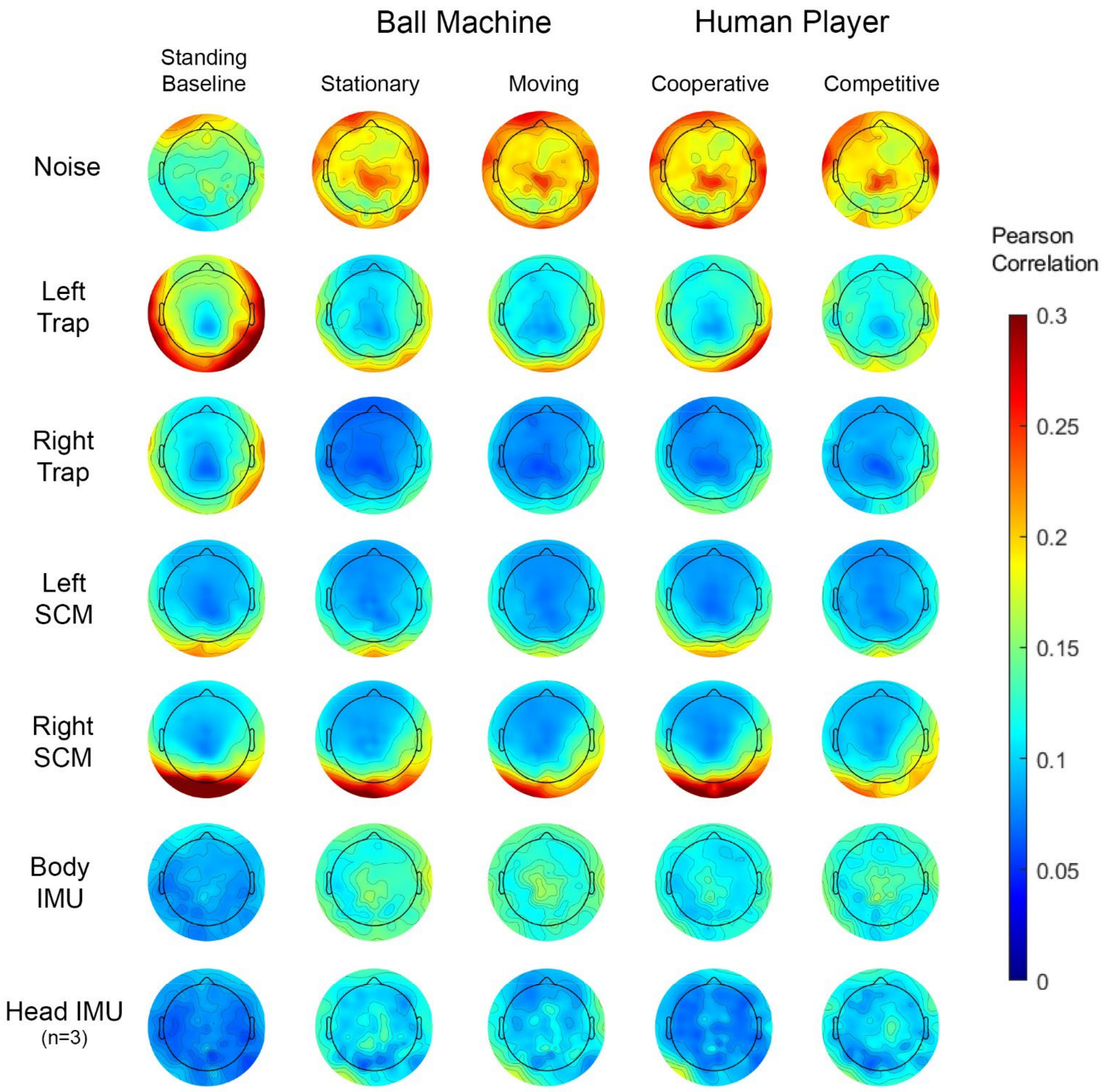

SUPPLEMENTARY FIGURE S3. **Group average time series correlations of individual scalp electrodes with paired noise electrodes (row 1), left and right trapezius (trap) muscle (rows 2 and 3), left and right sternocleidomastoid muscles (row 4 and 5), and resultant body IMU acceleration (row 6) with n=20 participants. The scalp electrode correlation with the resultant head IMU acceleration is shown in row 7 with n=3 participants. Each column shows a different condition. Pearson's R was converted to Fisher's Z for all participants and all conditions, averaged together, and converted back to Pearson's R.**
