# Supplementary material for: Characterizing and Removing Artifacts Using Dual-Layer EEG during Table Tennis
Source: Sensors (Basel). 2022 Aug 5;22(15):5867. doi: 10.3390/s22155867 (PMC9371038; doi:10.3390/s22155867)
Supplement: Supplementary file 1 [file sensors-22-05867-s001.zip › SupplementaryFigure_S5.pdf]

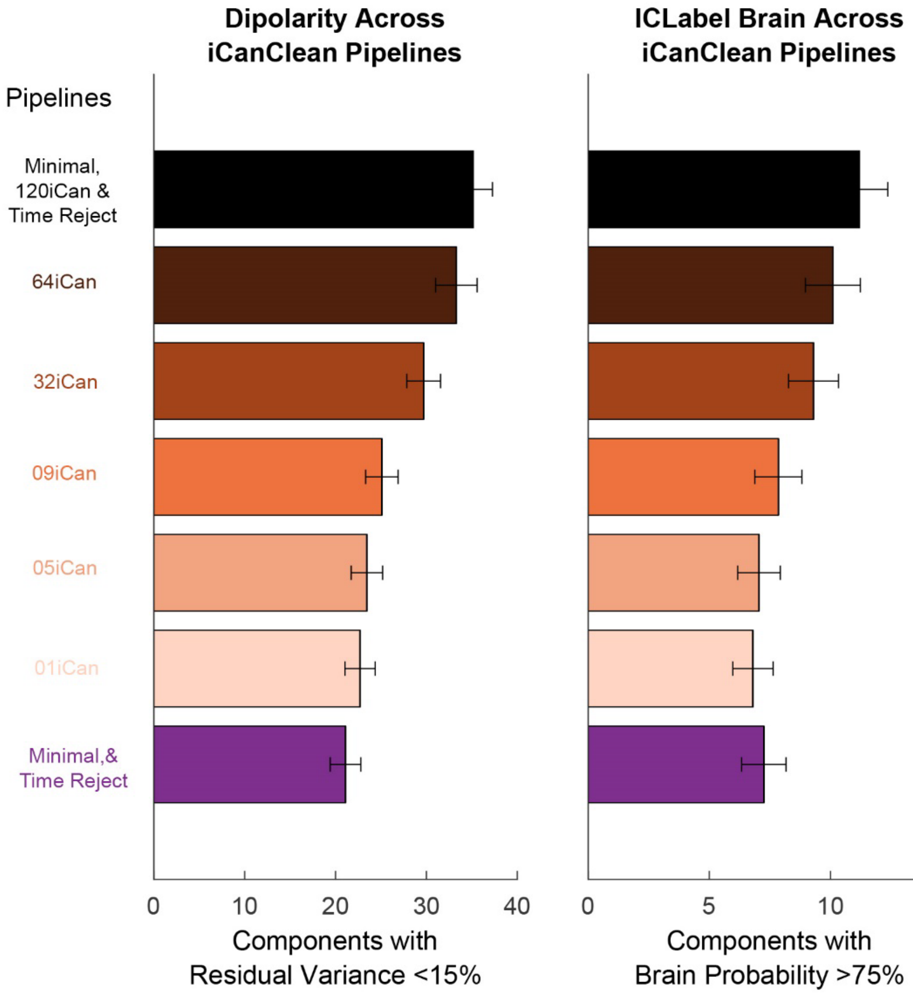

SUPPLEMENTARY FIGURE S5. **Quantitative assessment of the different iCanClean pipelines that use a different number of noise electrodes. A) Mean +/- SEM of dipolarity shows the quality of the ICA decomposition. Dipolarity is measured as the number of components with residual variance less than 15% after fitting dipoles with a three-layer boundary element model of the standard MNI brain template. B) Mean +/- SEM of the number of brain components with a brain probability > 75% from ICLabel. The *Minimal, 120iCan & Time Reject* pipeline (black) uses all 120 noise electrodes to clean scalp electrodes with canonical correlation analysis after minimal cleaning and with subsequent time window rejection. *64iCan – 01iCan* pipelines (brown gradients) use subsets of noise electrodes that are evenly spaced around the head. The iCanClean is applied after minimal cleaning and with subsequent time window rejection. The *Minimal & Time Reject* pipeline (purple) is equivalent to "0" noise electrodes used in iCanClean.**
