# Supplementary material for: Characterizing and Removing Artifacts Using Dual-Layer EEG during Table Tennis
Source: Sensors (Basel). 2022 Aug 5;22(15):5867. doi: 10.3390/s22155867 (PMC9371038; doi:10.3390/s22155867)
Supplement: Supplementary file 1 [file sensors-22-05867-s001.zip › SupplementaryFigure_S8.pdf]

Minimal, 120iCan, & Time Reject Pipeline

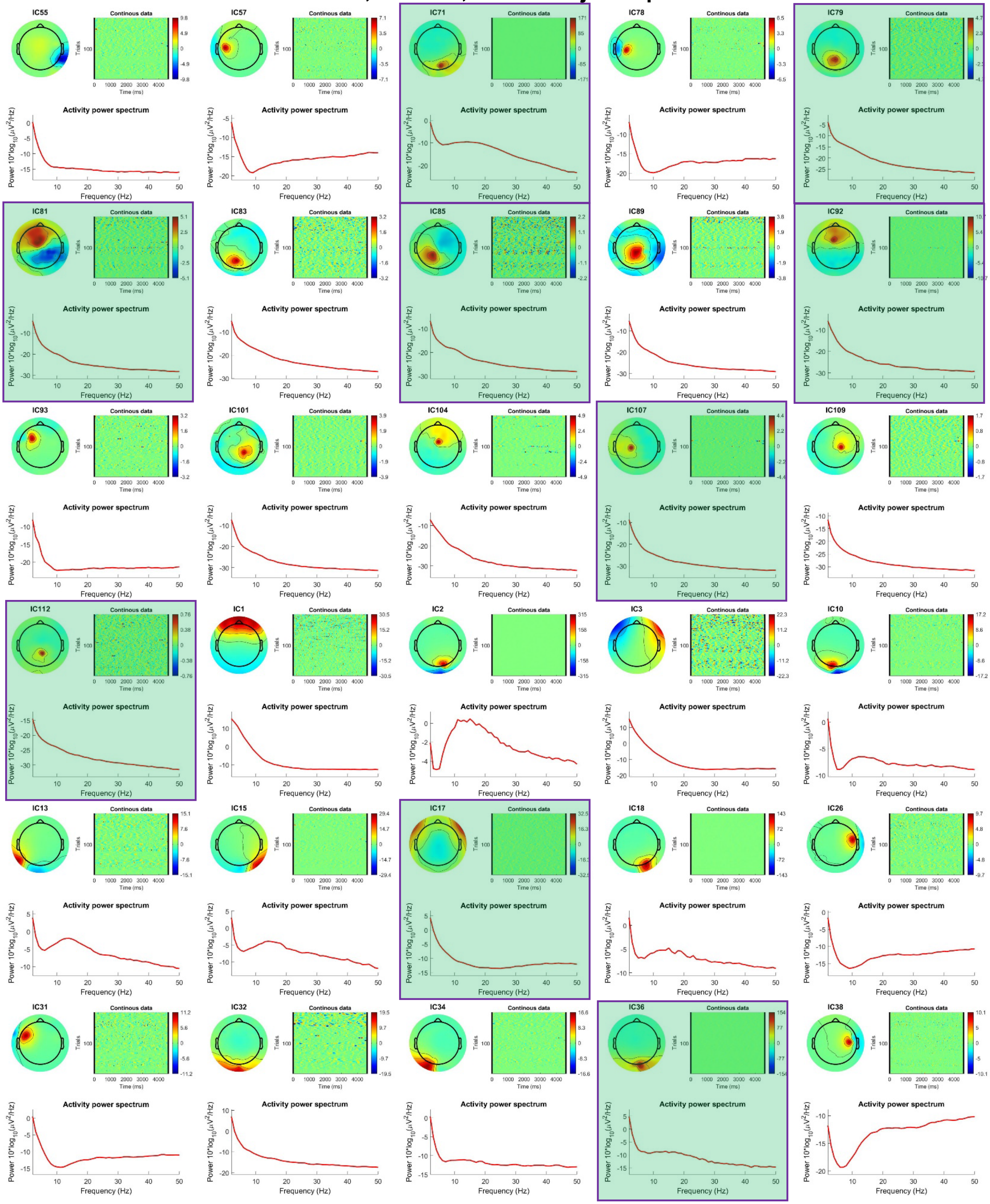

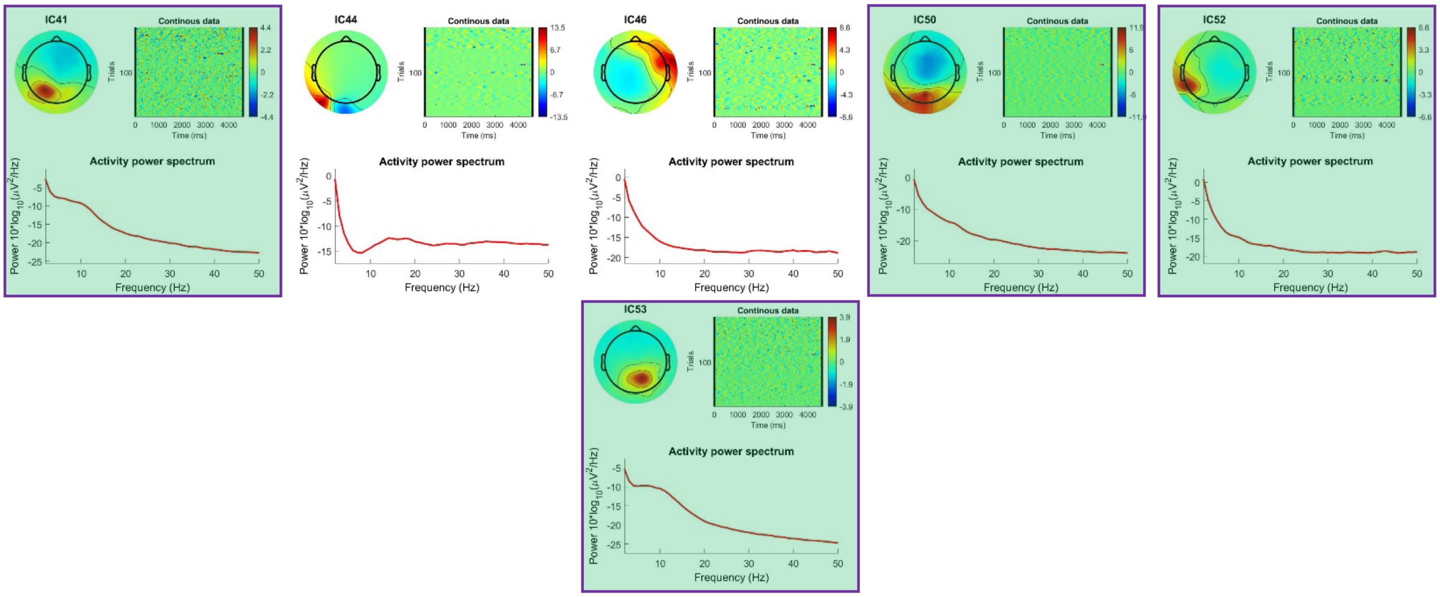

SUPPLEMENTARY FIGURE S8. Individual components from a single participant that passed the dipolarity metric (residual variance <=15%) after the Minimal, 120iCan & Time Reject Pipeline. Dipolar components labeled as >=75% brain by ICLabel are shaded in green.
